# The Paris Congress for the Study of Tuberculosis

**Published:** 1888-09

**Authors:** 


### THE PARIS CONGRESS FOR THE STUDY OF TUBERCULOSIS.

The names of the physicians and veterinarians who took an active part in the recent congress for the study of tuberculosis, which was held at Paris on July 25 to 31, 1888, are mainly French. Denmark, Scotland, and Egypt are other nations represented.

Some of the work reported is simply confirmatory of work and opinions long since placed before the profession; some of it is new, and much of the reports consists largely of expressions of opinion upon facts long ago generally accepted.

Of the various subjects discussed by the congress, of great interest and importance, one is the question of the relative frequency of the occurrence of tuberculosis among the lower animals, and of its transmission from them to man. M. Bang, of Copenhagen, contended that milk from tuberculous cows contains no bacilli of tuberculosis unless the milk glands are tuberculous, and that the tumefaction which the localized infiltration causes in the udder can be detected easily and early.

M. Grissonnauche claimed that from the first, pulmonary tuberculosis in cattle is characterized by tumefaction of the retropharyngeal glands ; there is interrupted respiration, and that a harsh friction sound is heard in auscultation. The cough is short and may be induced by sharp percussion blows upon the thorax, which evidently cause pain.

It was conceded that the degree of heat usually employed in cooking beef is not sufficient to kill the bacilli. It was admitted that the danger of infection to man through eating tuberculous beef is very slight, in Paris about six in one thousand, but nevertheless there were only three votes against a resolution which declared that all animals affected in any way with tuberculosis should be seized and condemned as unfit for food.

A very interesting and able discussion of the relation of animal tuberculosis to consumption in man will be found at page 43 of the July number of the JOURNAL AND EXAMINER in a report of the proceedings at a recent meeting of The Medico-Chirurgical Society of Edinburgh.

At the Paris congress the opinion was expressed that all cow's milk should be boiled before being given to infants.

The custom of drinking fresh blood and eating raw meat among consumptives was strongly condemned because of the danger of infection from these sources.

M. Cornil, in a paper on "Tuberculosis of Mucous Surfaces," said that the discovery that the tubercle-bacillus can penetrate the unbroken mucous membrane is one of extreme importance. He reported a case of tuberculosis of the cervix in a woman who had no other tubercular lesions. He introduced tubercular matter into the vaginæ of several guinea-pigs, and later found upon examination that the corresponding uteri were tuberculous.

M. Salles read a communication in which he reports the finding of a new microbe in the lungs of phthisical patients, which is distinct and separate, may be grown in pure cultures, and which, when injected into the circulation of rabbits produces death, with uniform and characteristic symptoms.

M. La Forre reported that as the result of investigations upon the subject he had concluded that the influence of a tuberculous father is greater than that of a mother similarly affected.

M. De Brun, of Beyrut, Syria, in a communication on "The Antagonism Between Malarial Fever and Tuberculosis," affirmed his belief in such an antagonism. In his opinion tuberculosis is a disease of communities which are drained and in good sanitary condition. Sometimes one is boldest in expressing opinions about things of which he knows least, but nevertheless, and although M. De Brun's colleague, M. Piot, of Cairo, agreed with him, and the report mentions no attempts at refutation of such ideas, this seems like a bit of careless observation and worse deduction.

The president of the congress, M. Chauveau, in the course of the discussion of the subject, said, that in his opinion the dangers of tubercular inoculation through vaccination against variola, are so infinitesimal in comparison with the benefits of that procedure that they are not worthy of consideration.

M. Arloing reported unsuccessful attempts to secure immunity from inoculations of tubercular bacilli, by means of inoculations of both material from "scrofulous" glands, and of typhoid bacilli.

As the result of recent observation, M. Cornil and M. Toupet reaffirm the already fully established observation that all pathological tubercular tumors are not necessarily evidence of the disease tuberculosis.

This meeting was evidently one of interest and importance, notwithstanding the fact that much of the ground which was discussed has long since been amply and conclusively cultivated by their Teutonic neighbors.

